# Concurrent Associations of Physical Activity and Screen-Based Sedentary Behavior on Obesity Among US Adolescents: A Latent Class Analysis

**DOI:** 10.2188/jea.JE20150068

**Published:** 2016-03-05

**Authors:** Youngdeok Kim, Tiago V. Barreira, Minsoo Kang

**Affiliations:** 1 Department of Health, Exercise, and Sport Sciences, Texas Tech University, Lubbock, Texas, USA; 2 School of Education, Syracuse University, Syracuse, NY, USA; 3 Department of Health and Human Performance, Middle Tennessee State University, Murfreesboro, Tennessee, USA

**Keywords:** screen time, exercise, BMI, YRBS

## Abstract

**Background:**

Independent associations of physical activity (PA) and sedentary behavior (SB) with obesity are well documented. However, little is known about the combined associations of these behaviors with obesity in adolescents. The present study examines the prevalence of concurrent levels of PA and SB, and their associations with obesity among US adolescents.

**Methods:**

Data from a total of 12 081 adolescents who participated in the Youth Risk Behaviors Survey during 2012–2013 were analyzed. A latent class analysis was performed to identify latent subgroups with varying combined levels of subjectively measured PA and screen-based SB. Follow-up analysis examined the changes in the likelihood of being obese as determined by the Center for Disease Control and Prevention Growth Chart between latent subgroups.

**Results:**

Four latent subgroups with varying combined levels of PA and SB were identified across gender. The likelihood of being obese was significantly greater for the subgroups featuring either or both Low PA or High SB when compared with High PA/Low SB across genders (odds ratio [OR] ranges, 2.1–2.7 for males and 9.6–23.5 for females). Low PA/High SB showed the greater likelihood of being obese compared to subgroups featuring either or both High PA and Low SB (OR ranges, 2.2–23.5) for female adolescents only.

**Conclusions:**

The findings imply that promoting sufficient levels of PA while reducing SB should be encouraged in order to reduce obesity risk among adolescents, particularly for males. The risk of obesity for female adolescents can be reduced by engaging in either high levels of PA or low levels of SB.

## INTRODUCTION

Obesity is a global pandemic that has been identified as one of the leading causes of preventable morbidity and mortality.^1^^–^^3^ The prevalence of obesity in adolescents has increased significantly in the last few decades,^4^ and this may directly lead to a future risk of developing chronic diseases, such as diabetes or metabolic syndrome later in life.^5^^,^^6^ A body of literature has identified physical activity (PA) as a potential modifiable lifestyle behavior that can reduce risk of obesity (and improve health profiles) among adolescents.^7^^,^^8^ Specific PA guidelines have been established to promote PA in youth (eg, ≥60 minutes of moderate and vigorous-intensity PA [MVPA] per day with ≥3 days of muscle-strengthening PA).^9^

Recently, the environmental and behavioral changes in modern society have extended a paradigm of health determinants to sedentary behavior (SB),^10^ which are characterized by a prolonged sitting or reclining posture requiring low level of energy expenditure (<1.5 METs), such as watching TV or using a computer.^11^^,^^12^ A great deal of evidence has been accumulated demonstrating SB as a health risk factor associated with a variety of chronic diseases across the lifespan.^13^ Specifically, excessive exposure to screen-based media is significantly associated with weight gain and increased risk of developing obesity among adolescents.^14^^,^^15^

PA and SB have become the major focus areas for preventing and explaining the risk of obesity among adolescents. Although some research has shown an interdependent relationship between these two behaviors, suggesting a possible tradeoff between one and the other,^16^^,^^17^ it is generally understood that PA and SB are distinct behaviors with different internal/external determinants^18^ that may explain unique variance of obesity risk.^17^^,^^19^^,^^20^ This line of thought has led researchers to examine the potential combined effects of PA and SB on the risk of obesity.^21^^–^^24^ To date, although direct comparison of studies is challenging due to variations in measurement of PA and SB (ie, subjective vs objective measurements), results have been unclear, with some studies showing additive effects,^22^^,^^24^ some showing independent effects,^20^ and others showing effects only for PA.^25^^,^^26^

One factor that might lead to inconsistent results may be the way in which PA and SB are combined to identify concurrent levels of PA and SB. A majority of large-scale observational studies, in which subjective measures of PA and SB are commonly preferred, have used a variable-centered approach that directly combines one or few implicit variables of PA and SB (eg, meeting aerobic PA guidelines and being in a low quartile of TV time) rather than relying on empirical evidence.^27^ This approach may introduce redundant classification errors, which may not be comprehensive enough to fully reflect various components of PA and SB simultaneously. Such limitations can be potentially overcome using latent class analysis (LCA), a person-centered approach identifying latent subgroups of a population with varying response patterns of multiple observed variables. LCA has been successfully employed in a previous PA study^27^; however, that study failed to adequately control for the possible influence of covariates on estimating the latent subgroups and in evaluating links with obesity in LCA model, which might produce biased estimates of population parameters.

The overarching goal of this study was to develop and examine a comprehensive LCA model examining the concurrent associations of PA and SB with obesity among United States (US) adolescents. Specific goals were to identify latent subgroups with varying concurrent patterns of PA and screen-based SB and to examine the association of latent subgroups with different level of PA and screen-based SB with obesity status among a national representative sample of US adolescents.

## METHODS

### Survey data and study sample

Data for this study came from the 2013 national Youth Risk Behavior Survey (YRBS), a biannual cross-sectional survey conducted by the Center for Disease Control and Prevention (CDC). The 2013 YRBS employed a three-stage cluster sampling design to obtain a national representative sample of adolescents in 9th to 12th grades attending public and private schools in the United States. In the first stage of sampling, the primary sampling units (PSUs; ie, counties) were organized into 16 strata based on their metropolitan statistical area status (ie, urban and rural) and the percentages of minority (black and Hispanic) students in the PSUs. The 54 PSUs were sampled with probability proportional to the total size of school enrollment in the PSU. In the second stage of sampling, secondary sampling units (SSUs; ie, schools) were categorized into two strata based on the size of schools. 193 schools with any of grades 9–12 were selected using probability proportional to school enrollment size. In the third stage of sampling, one class per grade was selected in each SSU (two classes per grade in SSUs with high minority enrollment) by varying selection methods from school to school (eg, random sampling, selecting from required courses such as English, or selecting during a particular time of day such as first or second period classes). All students in a sampled classroom were eligible for the survey.

The survey is primarily used to monitor a variety of health risk behaviors among youth, including PA and screen-based SB, using a standard questionnaire. Local parental permission was obtained from the participating schools prior to survey administration, and the protocol of the national YRBS was approved by the CDC’s Institutional Review Board. The response rates at the school and student levels were 77% and 88%, respectively, and adjustment for student and school non-responses was made when calculating the weights of students in participating schools. The detailed protocol of the 2013 national YRBS can be found elsewhere.^28^

A total of 13 583 adolescents completed a questionnaire in the 2013 national YRBS. Of these, the adolescents who provided valid responses on study variables were included. Those with missing values in gender, grade, body mass index (BMI), and three questions asking about healthy diet behaviors were excluded (*n* = 1460). In addition, 42 adolescents with ≥3 missing values among five questions asking about PA and screen-based SB were further excluded from the analysis. The final analytic sample consisted of 12 081 adolescents (6109 males), which represents 88.94% of the original sample.

### Measures

#### Self-reported PA and screen-based SB

The adolescents were asked to disclose the frequency of PA behaviors, including MVPA, sports team participation (STP), and muscle-strengthening exercise (MSEx). MVPA was determined by the question asking the number of days they had been physically active (ie, any kind of PA that increased heart rate and made breathe hard some of the time) for a total of at least 60 minutes per day during the past 7 days. The adolescents were dichotomized into either sufficient MVPA (S-MVPA) or insufficient MVPA (I-MVPA) categories based on the PA guideline for youths (≥60 minutes of MVPA per day).^9^ The number of sports teams (in the school or community group) in which the adolescents participated during the past 12 months was used for categorizing adolescents into either Active-STP (≥1 STP/year) or No-STP categories. In addition, the number of days engaging in MSEx (eg, push-ups, sit-ups, or weight lifting) during the past 7 days was obtained for categorizing adolescents into either sufficient-MSEx (S-MSEx) or insufficient-MSEx (I-MSEx) categories in accordance with the PA guideline for youths (≥3 days a week).^9^

Two questions asking about screen-based SBs provided the number of hours watching TV and using a computer for non-school work on an average school day (eg, time spent playing video or computer games; using all other screen-based technologies, such as smartphone or tablet; or Internet-related activities, such as social networking). Excessive exposures to TV and computer were determined using a criteria of ≥3 h per day for each behavior, respectively.^29^^,^^30^

#### Body mass index

Body mass index (BMI) calculated using self-reported height (cm) and weight (kg) was used to determine obesity. Adolescents were considered obese if BMI (kg/m^2^) was at or above 95th percentile according to the CDC sex-specific BMI-for-age growth chart.^31^

#### Study covariates

Demographic characteristics, including gender, grade (9th–12th), and race/ethnicity (non-Hispanic white, non-Hispanic black, Hispanic, or others) were obtained. In addition, information on healthy dietary behaviors was obtained from the questions asking about the frequency of having breakfast, consuming fruits and vegetables, and drinking soda during the past 7 days, as these diet behaviors may be confounding factors influencing the relationship of PA and screen-based SB with obesity.^32^

### Statistical analysis

LCA is a family of finite-mixture models representing the population heterogeneity by unobservable subpopulations. We first fitted a series of unconditional LCA models with differing number of latent subgroups (*k*; 1 through 6) in order to identify the model that best represents the heterogeneity of response patterns of PA and screen-based SB across a given number of latent subgroups. The model with the best model-data fits was determined using 1) the relative fit indices, including the Akaike Information Criterion (AIC), Bayesian Information Criterion (BIC), and sample size adjusted BIC (SABIC), where a lower value indicates a better model; 2) Lo-Mendell-Rubin adjusted likelihood ratio test (LMR-LRT), which compares the models with *k* and *k* − 1 latent subgroups; and 3) average classification probability (ACP) ranging between 0 and 1, in which a higher value indicates greater certainty in classification. In particular, BIC was preferred when comparing models based on results from a recent simulation study,^33^ and the practical interpretation of latent subgroups was also considered.^34^

The follow-up conditional LCA model with a distal outcome was fitted to examine 1) the likelihood of being classified as a respective latent subgroup based on grade and race/ethnicity; and 2) the likelihood of being obese based on a latent subgroup membership after controlling for grade, race/ethnicity, and healthy diet behaviors. All LCA analyses were stratified by gender to obtain gender-specific estimates, as there are gender disparities in PA and screen-based SB among adolescents.

LCA models were weighted for the three-stage sampling design of the 2013 national YRBS to obtain the population parameters of US adolescents. A robust full-information maximum likelihood algorithm was used for parameter estimations in order to account for missing responses in PA and screen-based SB. SAS version 9.3 (SAS Institute, Cary, NC, USA) and Mplus version 7.2 (Muthén & Muthén, Los Angeles, CA, USA) were used for data management and LCA analyses.

## RESULTS

### Descriptive statistics

Table 1 presents the descriptive statistics of study variables among US adolescents. There were significant gender disparities in the prevalence of PA and healthy dietary behaviors: male adolescents were more likely to be physically active and to have healthy dietary behaviors than female adolescents. In particular, more than half of US adolescents had either S-MVPA (57.6%; SE = 0.9), Active-STP (59.8%; SE = 1.3), or MSEx (57.8%; SE = 2.2). However, the prevalence of obesity was also greater for male adolescents (16.6%; SE = 0.8) compared to their female counterparts (10.8%; SE = 0.6). Additionally, no gender differences in the prevalence of screen-based SBs were observed.

**Table 1.** Descriptive statistics of study variables among US adolescents

	Total (*n* = 12 081)		Male (*n* = 6109)		Female (*n* = 5972)		*P*-value^a^
		
	%	SE	%	SE	%	SE	
Grade							0.6
9th	26.8	0.6	27.0	0.8	26.7	0.8	
10th	25.5	0.6	26.0	0.9	25.0	0.8	
11th	24.0	0.5	23.7	0.5	24.4	0.7	
12th	23.6	0.6	23.3	0.8	24.0	0.7	
Race/ethnicity							0.7
Non-Hispanic white	56.4	3.5	56.8	3.5	56.1	3.7	
Non-Hispanic Black	13.2	1.8	12.8	1.8	13.6	1.9	
Hispanic	20.3	2.2	20.3	2.3	20.3	2.3	
Others	10.1	1.1	10.2	1.1	10.1	1.1	
Breakfast consumption							<0.001
All 7 days	38.1	0.8	42.1	1.0	34.1	1.2	
≤6 days	61.9	0.8	57.9	1.0	65.9	1.2	
Fruit and vegetable consumption							<0.001
All 7 days	22.1	0.8	24.2	0.9	20.1	1.0	
≤6 days	77.9	0.8	75.8	0.9	79.9	1.0	
Soda consumption							<0.001
0 times/day	22.0	1.0	19.4	0.9	24.4	1.2	
≥1 times/day	78.0	1.0	80.6	0.9	75.6	1.2	
MVPA							<0.001
S-MVPA (≥60 minutes/day)	47.6	1.0	57.6	0.9	37.8	1.4	
I-MVPA (<60 minutes/day)	52.3	1.0	42.3	0.9	62.1	1.4	
Missing	0.1	0.0	0.1	0.1	0.1	0.0	
STP							<0.001
Active-STP (≥1 STP)	54.0	1.2	59.8	1.3	48.4	1.4	
No-STP (0 STP)	45.1	1.1	39.6	1.3	50.6	1.3	
Missing	0.9	0.5	0.7	0.4	1.0	0.6	
MSEx							<0.001
S-MSEx (≥3 day)	48.5	1.9	57.8	2.2	39.4	1.9	
I-MSEx (<3 day)	44.5	1.7	35.2	1.5	53.6	1.9	
Missing	7.0	3.0	7.0	3.1	7.0	3.0	
TV hours							0.6
≥3 hours/day	31.8	1.0	32.0	1.1	31.7	1.3	
<3 hours/day	67.9	1.0	67.7	1.2	68.2	1.3	
Missing	0.3	0.1	0.3	0.1	0.2	0.1	
Computer hours							0.2
≥3 hours/day	41.0	1.1	41.7	1.1	40.3	1.4	
<3 hours/day	58.7	1.1	57.9	1.1	59.5	1.4	
Missing	0.3	0.1	0.3	0.1	0.2	0.1	
Obese^b^							<0.001
Yes (≥95th percentile)	13.7	0.6	16.6	0.8	10.8	0.6	
No (<95th percentile)	86.3	0.6	83.4	0.8	89.2	0.6	

MVPA, moderate and vigorous physical activity; I-MSEx, insufficient-MSEx; I-MVPA, insufficient-MVPA; MSEx, muscle-strengthening exercise; S-MSEx, sufficient-MSEx; S-MVPA, sufficient-MVPA; STP, sports team participation; SE, standard error; TV, television.

^a^Rao-Scott *x*^2^ test of independence between male and female.

^b^Obesity was determined using the sex-specific BMI-for-age growth chart.

All estimates are weighted by a three-stage cluster sampling design of 2013 national YRBS.

### Latent class analysis

The model-data fit indices for unconditional LGA models with differing number of latent subgroups are presented in Table 2. The results showed that the heterogeneity of response patterns of PA and screen-based SB was best represented by four latent subgroups with the lowest BICs (43 920.0 and 41 682.3 for males and females, respectively), relatively high ACP (0.75 for both genders), and practical interpretability of item-response patterns of each latent class.

**Table 2.** Determining the number of latent subgroups using the unconditional LGM

Latent Class	Log- likelihood	AIC	BIC	SABIC	ACP	LMR-LRT	

						2LL	*P*-value
Male							
1	−23 170.1	46 352.3	46 395.7	46 373.6	—	—	—
2	−22 018.4	44 062.9	44 150.4	44 109.1	0.89	46 723.3	<0.001
3	−21 890.3	43 820.5	43 955.3	43 891.7	0.77	42 497.1	<0.001
4^a^	−21 842.0	43 738.1	43 920.0	43 834.2	0.75	42 093.8	<0.001
*(4)*^b^	*(*−*21 239.5)*	*(42 587.0)*	*(42 949.7)*	*(42 778.1)*	*(0.76)*	*(41 383.0)*	*(<0.001)*
5	−21 822.1	43 712.3	43 941.3	43 833.3	0.70	41 945.4	<0.001
6	−21 811.9	43 705.8	43 982.1	43 851.8	0.65	41 888.1	<0.001
Female							
1	−21 989.5	43 991.1	44 031.3	44 012.3	—	—	—
2	−20 860.9	41 747.7	41 834.9	41 793.6	0.90	46 708.7	<0.001
3	−20 785.4	41 610.8	41 745.0	41 681.7	0.80	42 350.4	<0.001
4^a^	−20 723.6	41 501.1	41 682.3	41 596.5	0.75	42 170.9	<0.001
*(4)*^b^	*(*−*20 144.8)*	*(40 397.6)*	*(40 759.1)*	*(40 587.5)*	*(0.72)*	*(41 351.9)*	*(<0.001)*
5	−20 709.6	41 487.3	41 715.5	41 607.4	0.66	41 951.4	<0.001
6	−20 704.8	41 491.7	41 766.8	41 636.5	0.66	41 905.3	<0.001

ACP, average classification probability; AIC, the Akaike Information Criterion; BIC, the Bayesian Information Criterion; LMR-LRT, Lo-Mendell-Rubin adjusted likelihood ratio test; SABIC, sample size adjusted BIC; 2LL, 2 times the log-likelihood difference between *k* and *k* − 1 class models.

^a^The model with an optimal number of latent subgroups from the unconditional LGM.

^b^The model-data fit indices from the conditional LGM with covariates adjustment and distal outcome.

All estimates are weighted by a three-stage cluster sampling design of 2013 national YRBS.

The item-response probabilities for each PA and screen-based SB item across latent subgroups are presented in Table 3. Higher probabilities in PA items indicates higher likelihood of being physically active (ie, engaging in MVPA, STP, and MSEx); whereas higher probabilities in screen-based SB items (ie, TV hours and computer hours) indicates higher likelihood of being engaged in excessive levels of SB. The results highlighted that latent subgroups are best characterized as the High PA/High SB (probability ≥0.5 for all of PA and screen-based SB items), High PA/Low SB (probability ≥0.5 and <0.5 for PA and screen-based SB items, respectively), Low PA/High SB (probability <0.5 and ≥0.5 for PA and screen-based SB items, respectively), and Low PA/Low SB (probability <0.5 for all of PA and screen-based SB items). The largest portion of male adolescents was characterized as the High PA/Low SB (38.6%; SE = 1.4), followed by Low PA/Low SB (33.5%; SE = 0.9); while the Low PA/Low SB was the largest latent subgroup among females (33.0%; SE = 1.4), followed by Low PA/High SB (26.4%; SE = 1.4).

**Table 3.** The estimated item-response probabilities of PA and screen-based SB across the latent subgroups

	Male				Female			
	
	Latent Class 1	Latent Class 2	Latent Class 3	Latent Class 4	Latent Class 1	Latent Class 2	Latent Class 3	Latent Class 4
% (SE)	20.3 (1.3)	38.6 (1.4)	7.7 (0.5)	33.5 (0.9)	17.6 (1.1)	23.1 (1.2)	26.4 (1.4)	33.0 (1.4)
Latent Class Profile^a^	High PA/ High SB	High PA/ Low SB	Low PA/ High SB	Low PA/ Low SB	High PA/ High SB	High PA/ Low SB	Low PA/ High SB	Low PA/ Low SB
Physical activity								
S-MVPA (≥60 minutes/day)	0.8*	0.9*	0.1	0.2	0.7*	0.9*	0.0	0.2
Active-STP (≥1 STP)	0.8*	0.8*	0.4	0.3	0.6*	0.8*	0.3	0.4
S-MSEx (≥3 day/week)	0.9*	0.9*	0.1	0.2	0.7*	0.9*	0.1	0.2
Screen-based sedentary behaviors								
TV hours (≥3 hours/day)	0.7*	0.1	1.0*	0.2	0.6*	0.1	0.6*	0.1
Computer hours (≥3 hours/day)	0.6*	0.2	0.7*	0.48	0.6*	0.2	0.6*	0.3

Active-STP, active sports team participation; PA, physical activity; S-MVPA, sufficient moderate or vigorous physical activity; S-MSEx, sufficient muscle-strengthening exercise; SB, sedentary behavior; SE, standard error; TV, television.

^a^Latent class profile was determined based on the estimated probabilities of PA and screen-based SB for each group.

*Estimated probability ≥0.50 for a respective item.

All estimates are from the conditional LGA model adjusted by grades and race/ethnicity, and are weighted by a three-stage cluster sampling design of 2013 national YRBS.

Grade and race/ethnicity were all significant correlates influencing the likelihood of being classified as a respective subgroup compared to the High PA/Low SB subgroup (Table 4). Specifically, 10th–12th graders were more likely to be in the High PA/High SB subgroup compared to 9th graders for both male and female participants. Among females, Hispanic adolescents were less likely to be in the High PA/High SB subgroup (OR 0.7; 95% CI, 0.4–0.98) and more likely to be in the Low PA/Low SB subgroup (OR 2.2; 95% CI, 1.3–3.5) compared to non-Hispanic white adolescents.

**Table 4.** Correlates of latent class memberships with grades and race/ethnicity^a^

	High PA/High SB		Low PA/High SB		Low PA/Low SB	
		
	OR	95% CI	OR	95% CI	OR	95% CI
Males						
Grade						
9th	(ref)	—	(ref)	—	(ref)	—
10th	17.1	(8.8, 33.5)	12.33	(4.5, 33.5)	3.11	(1.7, 5.6)
11th	2.6	(1.7, 3.8)	2.14	(1.3, 3.5)	1.33	(0.98, 1.8)
12th	3.7	(2.5, 5.7)	1.74	(0.6, 5.0)	1.76	(1.3, 2.4)
Race/ethnicity						
Non-Hispanic white	(ref)	—	(ref)	—	(ref)	—
Non-Hispanic Black	1.0	(0.6, 1.6)	1.05	(0.6, 2.0)	1.22	(0.8, 1.9)
Hispanic	0.9	(0.6, 1.4)	1.11	(0.6, 2.1)	1.22	(0.9, 1.7)
Others	0.9	(0.6, 1.3)	1.51	(0.9, 2.6)	1.41	(1.0, 1.9)
Females						
Grade						
9th	(ref)	—	(ref)	—	(ref)	—
10th	11.0	(5.2, 23.1)	11.59	(4.9, 27.7)	1.34	(0.3, 5.3)
11th	4.9	(3.0, 8.0)	3.59	(2.1, 6.2)	1.28	(0.7, 2.3)
12th	2.8	(1.4, 5.9)	3.17	(1.8, 5.5)	1.45	(0.7, 2.9)
Race/ethnicity						
Non-Hispanic white	(ref)	—	(ref)	—	(ref)	—
Non-Hispanic Black	0.8	(0.5, 1.2)	0.73	(0.5, 1.1)	1.21	(0.8, 1.8)
Hispanic	0.7	(0.4, 0.98)	1.05	(0.6, 1.7)	2.16	(1.3, 3.5)
Others	0.6	(0.3, 1.3)	1.09	(0.6, 1.9)	2.51	(1.7, 3.8)

CI, confidence interval; OR, odds ratio; PA, physical activity; SB, sedentary behavior.

^a^High PA/Low SB was used as a reference group.

All estimates are from the conditional LGA model with covariate adjustment and distal outcome, and are weighted by a three cluster sampling design of 2013 national YRBS.

The findings for predicting the likelihood of being obese across latent subgroups after controlling for grade, race/ethnicity, and healthy dietary behaviors are presented in Table 5. Compared to the High PA/Low SB subgroup, all other subgroups were more likely to be obese for both genders. In particular, the Low/ PA/High SB showed highest odds of being obese (OR 4.7; 95% CI, 1.7–12.9 for males and OR 23.5; 95% CI, 4.3–127.7 for females), followed by the High PA/High SB and Low PA/Low SB subgroups for both genders.

**Table 5.** Comparisons of the likelihood of being obese across the latent subgroups with different patterns of PA and screen-based SB

	OR	95% CI	

		Lower	Upper
Male			
*(Reference: High PA/Low SB)*			
High PA/High SB	2.7	2.0	3.8
Low PA/High SB	4.7	1.7	12.9
Low PA/Low SB	2.1	1.5	3.0
*(Reference: Low PA/Low SB)*			
High PA/High SB	1.3	0.98	1.8
Low PA/High SB	2.3	0.8	6.3
*(Reference:* High PA/High SB*)*			
*Low PA/High SB*	1.7	0.6	4.6
Female			
*(Reference: High PA/Low SB)*			
High PA/High SB	10.8	1.7	67.2
Low PA/High SB	23.5	4.3	127.7
Low PA/Low SB	9.6	1.8	51.4
*(Reference: Low PA/Low SB)*			
High PA/High SB	1.1	0.6	2.2
Low PA/High SB	2.4	1.3	4.6
*(Reference: High PA/High SB)*			
Low PA/High SB	2.2	1.4	3.3

CI, confidence interval; OR, odds ratio; PA, physical activity; SB, sedentary behavior.

All estimates are from the conditional LGA model while controlling for grades, race/ethnicity, and healthy diet behaviors, and are weighted by a three cluster sampling design of 2013 national YRBS.

The likelihood of being obese was not significantly different between the High PA/High SB, Low PA/Low SB, and Low PA/High SB subgroups for males, while the Low PA/High SB subgroup had greater odds of being obese compared to the Low PA/Low SB (OR 2.4; 95% CI, 1.3–4.6) and High PA/High SB (OR 2.2; 95% CI, 1.4–3.3) subgroups for female adolescents.

## DISCUSSION

Using a national representative sample of US adolescents, we found that the heterogeneity of PA and screen-based SBs in adolescents can be best captured by four latent subgroups for both genders. Conceptually, it is reasonable to assume that the choice to engage in SB may decrease PA and vise-versa, as they are aligned at opposite ends of the behavioral spectrum.^16^ A growing body of literature, however, confirms the independent relationship between these two behaviors, with specific evidence for MVPA and SB,^35^ and our findings generally support this claim. In particular, our unique analytic approach focusing on intra-individual variations in multiple structured PA and SB indicators provided empirical evidence of the most likely subgroups with varying concurrent patterns of PA and SB among adolescents. The results revealed that the response probabilities for PA and SB indicators are likely interrelated within each domain but not across domains, yielding distinct and independent patterns of PA and SB across latent subgroups (High PA/Low SB, High PA/High SB, Low PA/High SB, and Low PA/Low SB).

The findings highlight gender disparity with regards to PA and SB. Gender differences in healthy behaviors have been continuously reported across all age groups. Specifically, it has been well-documented that female adolescents have lower levels of PA and higher levels of SB compared to male adolescents^27^^,^^36^^,^^37^; however, a preponderance of evidence has been accumulated for a single variable of PA and SB separately, limiting our understanding of possible gender disparity in concurrent prevalence of PA and SB among adolescents. The present findings extend the literature by describing the gender differences in the latent subgroups identified based on the combined patterns of PA and SB. The largest portion of male adolescents were categorized into the High PA/Low SB (38.6%; SE = 1.4) subgroup, while the Low PA/Low SB (33.0%; SE = 1.4) subgroup was the largest for female adolescents. More importantly, the Low PA/High SB subgroup, which is potentially the highest risk group for health, was significantly larger for females (26.4; SE = 1.4) compared to males (7.7%; SE = 0.5). Considering that PA and SB are important health determinants in later life,^14^ public efforts to develop and implement gender-specific intervention strategies to promote PA and reduce SB should be made.

As previously mentioned, the independent associations of PA and SB with obesity have been frequently examined among adolescents; however, findings are mixed and limited, in part, to extending our understanding of combined effects on the risk of obesity. For example, a study examining the independent relationships of objectively measured PA and self-reported TV hours with obesity among 2200 adolescents from 10 European cities demonstrated that excessive TV watching (>4 hours per day) was a significant predictor of increased likelihood of being obese even after adjusting for PA levels.^38^ Another study also reported a stronger association of screen time with overweight and obesity than PA among 2200 Australian adolescents.^19^ In contrast, PA has frequently been reported as the only risk factor of obesity among adolescents in several cross-sectional observations.^25^^,^^26^

The present findings provide empirical evidence to support the complexity of combined effects of PA and SB for explaining the risk of obesity in this population. The use of an advanced statistical method based on LCA allowed us to classify the population by the response patterns of multiple PA and screen-based SB indicators and to include the distal outcome of obesity directly into the model while adjusting for covariates, including gender, race/ethnicity, and healthy diet behaviors. For both genders, the High PA/Low SB subgroup showed significantly lower likelihood of being obese compared to other subgroups; however, the relative magnitudes of such effects were greater for females compared to male adolescents. This may imply that increasing PA and reducing SB are equally important to reduce obesity risk among adolescents, in that significant health benefits will likely be obtained by having healthy behaviors for both PA and SB, with greater likelihood for preventing obesity being expected for females than males. Our findings are generally aligned with those of a previous report that both low PA and excessive TV hours are important risk factors for being overweight in adolescents, with stronger associations observed in female adolescents.^22^

Our analyses also indicated that there could be some differences in the concurrent effects of PA and SB on obesity between genders. Specifically, the odds of being obese were not significantly different between the High PA/High SB, Low PA/High SB, and Low PA/Low SB subgroups for males, while female adolescents in the Low PA/High SB subgroup showed a significantly greater likelihood of being obese compared to their female counterparts in the Low PA/Low SB and High PA/High SB subgroups. These findings imply that for male adolescents to reduce the risk of obesity, which would be a significant health benefit, they must have both high levels of PA and low levels of SB. Pertaining to adolescent females, having either high levels of PA or low levels of SB may reduce risk of being obese to a greater degree than engaging in both low levels of PA and high levels of SB. Our findings demonstrating distinct combined associations of PA and SB with the risk of obesity across gender may also partially explain the prevalence of obesity being lower in female adolescents (10.8%) than in males (16.6%).

Taken together, these findings suggest that gender-specific PA and SB recommendations and intervention strategies might be necessary. Although it is difficult to make a concrete explanation of such gender differences due to the limited resources in this survey, one possible reason could be related to the differences in calories expended and consumed during the engagements of PA and SB, respectively, between genders.^14^ Energy imbalance is regarded as a common cause of obesity, and gender differences in the response of energy expenditure during daily PA have been previously reported.^39^ Moreover, the likelihood of having unhealthy foods while engaging in screen-based SB is reportedly increased, with female adolescents being more likely to consume unhealthy foods than males.^38^

The interpretation of the present findings should account for several limitations. First, the 2013 national YRBS is a cross-sectional survey that precludes assessment of the casual relationships among study variables. As noted above, a longitudinal study examining the trajectories of PA and SB in relation to time-variant determinants as well as the changes in obesity would be a promising way to better understand the complex nature of PA, SB, and the influence of those behaviors on risk of obesity among adolescents. Second, while our findings were controlled for demographic characteristics and healthy diet behaviors, we were not able to control for additional potential confounding factors, such as previous weight status, due to the limited data available in the YRBS. Third, the indicators of PA and SB are subjectively measured and are limited to address only some aspects of PA (ie, MVPA, STP, and MSEX) and SB (ie, watching TV and using a computer). This might lead participants to overestimate their actual levels of PA and SB, which are subject to recall bias.^40^ Furthermore, obesity status was determined based on self-reported height and weight. The objectively measured adiposity levels, PA, and SB across specific contexts would be warranted in future studies in order to strengthen the external validity of the findings.

In conclusion, US adolescents can be classified into four latent subgroups based on the response patterns of PA and screen-based SB. The estimated latent subgroups showed significantly different likelihoods of being obese between each other, indicating the complex associations of PA and SB with the risk of obesity. For both genders, both PA and SB are important lifestyle behaviors related to obesity. More specifically, the High PA/Low SB subgroup, who had the highest probability of compliance with current PA and screen-based SB recommendations, had significantly lowest odds of being obese, with greater odds for female adolescents than males. In addition, female adolescents may expect to have some health benefits by complying with either PA or SB recommendations, while male adolescents should be encouraged to comply with both PA and SB recommendations in order to achieve significant health benefits. The present findings imply the need for developing gender-specific PA and/or SB intervention strategies to maximize health benefits of reducing the risk of obesity among adolescents.
